# Phosphorus-bearing molecules PO and PN at the edge of the Galaxy

**DOI:** 10.1038/s41586-023-06616-1

**Published:** 2023-11-08

**Authors:** L. A. Koelemay, K. R. Gold, L. M. Ziurys

**Affiliations:** 1 Department of Chemistry, University of Arizona, Tucson, AZ USA; 2 Department of Astronomy, and Steward Observatory, University of Arizona, Tucson, AZ USA

**Keywords:** Interstellar medium, Stellar evolution

## Abstract

Despite its importance in planet formation and biology^1^, phosphorus has been identified only in the inner 12 kpc of the Galaxy^2–19^. The study of this element has been hindered in part by unfavourable atomic transitions^2,4,20^. Phosphorus is thought to be created by neutron capture on ^29^Si and ^30^Si in massive stars^20,21^, and released into the interstellar medium by Type II supernova explosions^2,22^. However, models of galactic chemical evolution must arbitrarily increase the supernovae production^23^ to match observed abundances. Here we present the detection of gas-phase phosphorus in the Outer Galaxy through millimetre spectra of PO and PN. Rotational lines of these molecules were observed in the dense cloud WB89-621, located 22.6 kpc from the Galactic Centre^24^. The abundances of PO and PN in WB89-621 are comparable to values near the Solar System^25^. Supernovae are not present in the Outer Galaxy^26^, suggesting another source of phosphorus, such as ‘Galactic Fountains’, where supernova material is redistributed through the halo and circumgalactic medium^27^. However, fountain-enriched clouds are not found at such large distances. Any extragalactic source, such as the Magellanic Clouds, is unlikely to be metal rich^28^. Phosphorus instead may be produced by neutron-capture processes in lower mass asymptotic giant branch stars^29^ which are present in the Outer Galaxy. Asymptotic giant branch stars also produce carbon^21^, flattening the extrapolated metallicity gradient and accounting for the high abundances of C-containing molecules in WB89-621.

## Abstract (web summary)

We report the presence of gas-phase phosphorous at the edge of the Galaxy and suggest it is produced by neutron-capture processes in lower mass asymptotic giant branch stars.

## Main

In the Outer Galaxy, roughly defined as more than 16 kpc from the Galactic Centre, molecular clouds are typically small and sparse^30^, resulting in a lower star formation rate^24^. Supernovae are also extremely rare in this part of the Galaxy, with none known beyond 13 kpc (ref. ^26^). Elemental abundance gradients estimated for carbon, oxygen and nitrogen support a decline in star formation in the Outer Galaxy. The gradients suggest a decrease in abundances by factors of 10, 3 and 5, respectively, for C, O and N, from 8.5 to 23 kpc (ref. ^31^). However, these studies do not typically characterize the metallicity of the Galaxy beyond 13–16 kpc (refs. ^31,32^), such that extrapolation beyond these distances is highly uncertain.

**Fig. 1:**
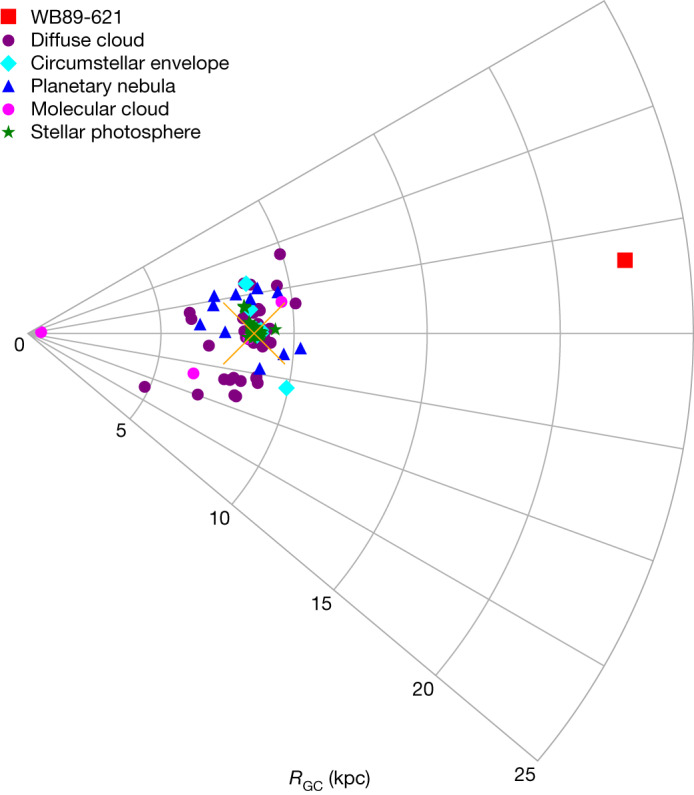
Currently known Galactic distribution of phosphorous. A graphic illustrating the Galactic distribution of phosphorus in the range 0–25 kpc from the Galactic Centre, as identified from stellar and interstellar observations. Detections by atomic transitions in stellar photospheres are denoted by green stars (from ref. ^2^ and references therein), in diffuse clouds by purple circles (ref. ^16^ and those therein^17–19^), and in planetary nebulae by blue triangles^6–15^, while magenta circles and cyan diamonds mark the identifications in molecular clouds and circumstellar envelopes from molecular lines^25,34–36,40^. The red square shows the position of WB89-621. The orange X indicates the solar system. The APOGEE survey detections are not shown per possible contamination. WB89-621 is the only known source of phosphorus in any form beyond 12 kpc.

Recent observations of so-called Galactic edge clouds have demonstrated that dense gas at large galactocentric distances (approximately 13–24 kpc) contains unusual amounts of gas-phase carbon-bearing molecules such as methanol^33^. Therefore, we conducted a search for the phosphorus-bearing molecules PO and PN in one of these star-forming regions, WB89-621 (right ascension (α) = 05 h 17 min 13.3 s, declination (δ) = 39°22′14″ (J2000.0)), located at a galactocentric radius of 22.6 kpc (ref. ^24^). PN and PO had previously been found in molecular clouds, including Orion-KL, AFGL 5142 and W51 (refs. ^25,34–36^), as well as in circumstellar envelopes of evolved stars. However, these molecular observations have been limited to less than 10 kpc of the Galactic Centre (Fig. 1). Millimetre-wave observations of WB89-621 were conducted with the Arizona Radio Observatory (ARO) 12 m telescope and the Institut de Radioastronomie Millimétrique (IRAM) 30 m telescope. The *J* = 3 → 2 and *J* = 2 → 1 transitions of PN at 2 and 3 mm were identified, as well as the four hyperfine/lambda-doubling components of the *J* = 2.5 → 1.5 transition of PO (Table 1).

**Table 1:** Observations of PN (X^1^Σ^+^), PO (X^2^Π_r_) and other molecules towards WB89-621

Molecule	Transition^a^	Frequency (MHz)	*T*_A_^*^ (mK)	∆*V*_1/2_ (km s^−1^)^c^	*V*_LSR_ (km s^−1^)	*f*(X/H_2_)^d^
PN	*J* = 2 → 1	93,979.8	10 ± 2	1.7 ± 0.6	−25.1 ± 0.6	3.0 ± 1.6 × 10^−12^
	*J* = 3 → 2^b^	140,967.7	8 ± 3	1.2 ± 0.5	−25.4 ± 0.4	
PO	*J* = 2.5 → 1.5 *F* = 2 → 1, e	109,045.4	7 ± 2	1.6 ± 0.5	−25.3 ± 0.6	2.0 ± 1.1 × 10^−11^
	*J* = 2.5 → 1.5 *F* = 3 → 2, e	108,998.4	8 ± 2	1.4 ± 0.5	−25.4 ± 0.6	
	*J* = 2.5 → 1.5 *F* = 2 → 1, f	109,281.2	6 ± 2	1.6 ± 0.5	−25.9 ± 0.6	
	*J* = 2.5 → 1.5 *F* = 3 → 2, f	109,206.2	8 ± 2	1.4 ± 0.5	−25.4 ± 0.6	
N_2_H^+^	*J* = 1 → 0 *F*_1_, *F* = 1,2 → 1,2	93,171.9	85 ± 2	2.3 ± 0.6	−25.4 ± 0.6	1.1 ± 0.3 × 10^−10^
	*J* = 1 → 0 *F*_1_, *F* = 2,3 → 1,2	93,173.8	127 ± 2	2.9 ± 0.6	−25.2 ± 0.6	
	*J* = 1 → 0 *F*_1_, *F* = 0,1 → 1,2	93,176.3	38 ± 2	1.8 ± 0.6	−25.3 ± 0.6	
CH_3_OH	*J*_τ_ = 8_0_ → 7_1_, A	95,169.4	33 ± 2	2.3 ± 0.6	−25.6 ± 0.6	1.1 ± 0.1 × 10^−9e^

^a^Measured with the IRAM 30 m unless indicated otherwise.

^b^Measured with the ARO 12 m.

^c^Linewidth at half maximum.

^d^Abundance relative to H_2_ (ref. ^24^).

^e^From ref. ^33^.

**Fig. 2:**
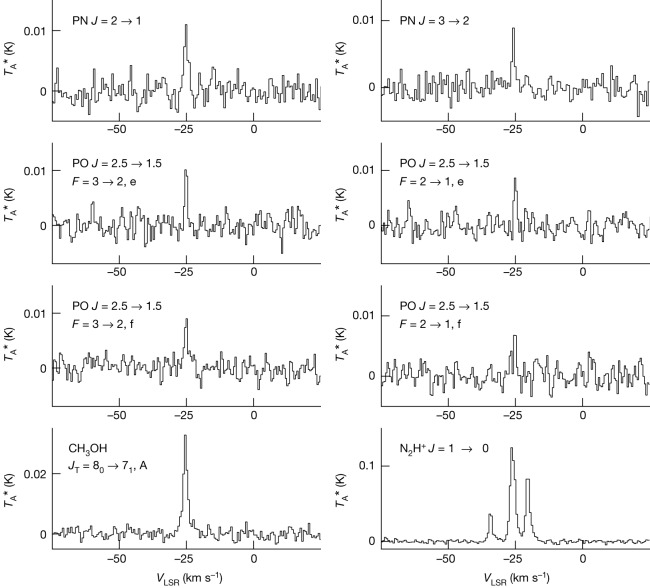
Spectra of molecular rotational transitions of PO, PN and other species in molecular cloud WB89-621. Spectra are plotted as intensity (*T*_A_*), in mK, versus *V*_LSR_, in kilometres per second. The top two panels show the *J* = 2 → 1 and *J* = 3 → 2 transitions of PN, followed by the four hyperfine/lambda-doubling components of the *J* = 2.5 → 1.5 transition of PO. The bottom two panels display the N_2_H^+^ *J* = 1 → 0 and CH_3_OH *J*_τ_ = 8_0_ → 7_1_, A transitions. Each panel includes the molecular species and quantum numbers corresponding to the rotational transition shown. The *J* = 2 → 1 line of PN (156 kHz spectral resolution) was measured with the 12 m antenna of the ARO, while all others were observed using the IRAM 30 m telescope (200 kHz spectral resolution).

The measured spectra of PN and PO in WB89-621 are shown in the top six panels in Fig. 2. The spectral profiles typically exhibit narrow linewidths of approximately 1.5 km s^−1^ and have a velocity with respect to the local standard of rest (*V*_LSR_) near −25.4 km s^−1^, characteristic of molecular material in WB89-621, as illustrated in the lower two panels of Fig. 2. Here spectra of the *J* = 1 → 0 transition of N_2_H^+^ and the *J*_τ_ = 8_0_ → 7_1_, A of CH_3_OH are shown. In the N_2_H^+^ features, nitrogen quadrupole hyperfine components are resolved. Note that aside from first-order baseline subtraction, no other data reduction or model dependent analysis has been done, unlike with stellar spectra^2^. Line parameters for all species, determined from Gaussian fits to the line profiles, are summarized in Table 1. The linewidths and LSR velocities are consistent among these molecules. Note that the PO and PN detections are separate measurements, and each give an independent assessment of the phosphorus abundance.

**Fig. 3:**
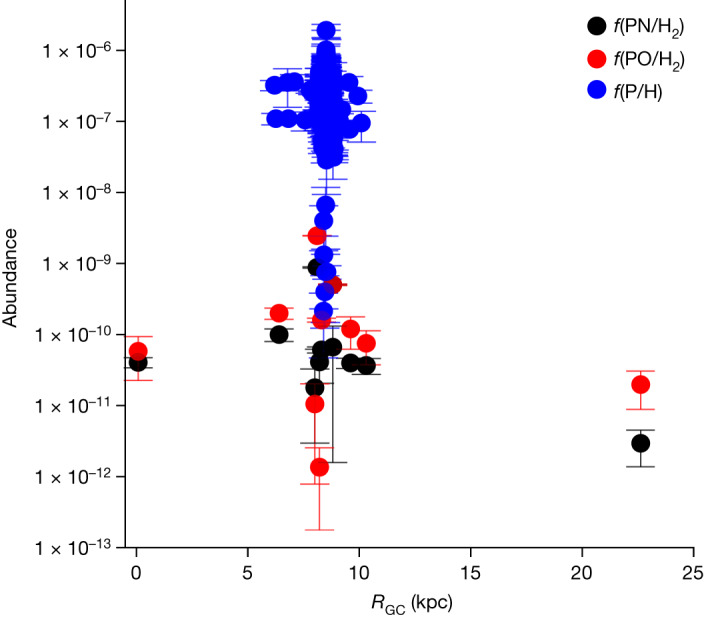
Abundances of PN and PO, as well as atomic phosphorus, as a function of distance from the Galactic Centre. Molecular abundances, relative to H_2_, are plotted with respect to *R*_GC_ (kpc) for AFGL 5142, G + 0.693-0.03, W3(OH), W51, L1157, Orion-KL^25^, B1-a, NGC 1333-IRAS 3, Ser SMM1, L723 (ref. ^36^) and WB89-621. Atomic abundances from sources in Fig. 1 are also displayed. Abundances of PO, PN and atomic P are shown in red, black and blue, with 3*σ* estimated uncertainties. From 8.5 to 22.6 kpc, abundances of PO and PN decrease by a factor of 1.5 and 2.3, respectively, showing little decrease in the Outer Galaxy.

The abundances of PN and PO relative to H_2_ were calculated to be 3.0 ± 1.6 × 10^−12^ and 2.0 ± 1.1 × 10^−11^, respectively. These values are comparable to those found in the solar neighbourhood, as shown in Fig. 3. A linear fit to the PO and PN abundances with respect to distance from the Galactic Centre (*R*_GC_) suggests a slight gradient—roughly a factor of 1.5 and 2.3 decrease in abundance from 8.5 to 22.6 kpc for PO and PN, respectively. The abundances of phosphorus, derived from limited observations of atomic lines in stellar photospheres (see ref. ^2^ and those therein), diffuse clouds^16–19^ and planetary nebulae^6–15^ (Fig. 1) are also shown in Fig. 3, plotted on the same galactocentric distance scale. These measurements cluster primarily between 6 and 10 kpc, with no values beyond 12 kpc (ref. ^7^). Extrapolating from a relatively small galactocentric range to across the Galaxy is problematic; a simplistic analysis suggests a drop in abundance by a factor of 10 between 8.5 and 22.6 kpc, but such an estimate may be misleading. Note that the solar abundance of phosphorus is [P/H] approximately 2.6 × 10^−7^ (ref. ^37^).

Because phosphorus is thought to be produced by supernovae^3^, and there are few, if any, supernovae beyond 13 kpc (ref. ^26^), another form of elemental enrichment must be occurring. One possibility is a ‘Galactic Fountain’, where supernovae eject gas into the halo/disk-halo interface, where it cools and subsequently ‘rains’ down on the disk^27^. Such fountains, however, are not known to operate beyond 15 kpc. Furthermore, the infall from a fountain creates distinct clouds^38^ with ‘forbidden velocities’ that do not follow the galactic rotation curve^27^. Given the location of WB89-621, the rotation curve^27,38^ would indicate *V*_LSR_ ≈ 0 km s^−1^, as opposed to the actual LSR velocity of −25.4 km s^−1^. Velocities less than 30 km s^−1^ can be accounted for by turbulence^38^. WB89-621 also resides roughly 300 pc (galactic latitude *b* = 0.8197°) above the Galactic midplane, while most fountain clouds have galactic latitudes greater than 3° and are clearly separated from the disk^27^. The origin of this phosphorus is also unlikely to be from extragalactic sources, such as the Large Magellanic Cloud. Although the outer regions of the Milky Way are thought to have interacted with the Large Magellanic Cloud, such dwarf galaxies are characterized by low metallicity^28^ and therefore are unlikely to cause phosphorus enrichment.

The source of phosphorus in the Outer Galaxy may be alternative routes in stellar nucleosynthesis. Because of the failure of the galactic chemical evolution models, it has been postulated that non-explosive massive stars or thermal-pulsing asymptotic giant branch stars (AGB) stars may be possible sources of this element^29^. The lower mass population of stars in the Outer Galaxy^39^ suggests an AGB origin. In such stars, a neutron excess can be produced in the H/He shell interface, where a pocket of ^13^C is generated from the addition of protons to ^12^C. An excess of neutrons is then created through the reaction ^13^C(α,*n*)^16^O that leads to overproduction of ^31^P and other elements. Models have been speculative about the phosphorus yields from AGB stars, ranging from ‘negligible’ to enhancements of 4–20, depending on stellar mass and metallicity^3,29^. The surprising number of phosphorus-bearing molecules observed in the circumstellar envelopes of AGB stars provide some additional evidence for this hypothesis^40^. There may be other possible reactions leading to phosphorus in AGB stars as well, such as proton addition to ^30^Si and α-capture on ^27^Al (ref. ^20^). Furthermore, it has been suggested that phosphorus may exhibit primary element behaviour over the metallicity range for [Fe/H] between −0.9 and 0.3 (ref. ^3^), meaning that it is generated by reactions directly from H and He. Extrapolating the gradient measured out to 20 kpc to 23 kpc (ref. ^41^), the [Fe/H] ratio in WB89-621 is −0.61. If primary behaviour is indeed attributable to this element, then its existence in the Outer Galaxy is perhaps expected.

AGB stars, which generate roughly half of all ^12^C (ref. ^21^), may also be responsible for the high abundances of C-containing molecules in the Outer Galaxy. Methanol (CH_3_OH), for example, has been observed in twenty edge clouds in the range 13–23 pc. The abundance of CH_3_OH has been found to remain fairly constant from 7 to 23 kpc (ref. ^33^), despite the predicted 10-fold decline in carbon over the same distances^31^. However, this elemental gradient is extrapolated from measurements in H ii regions that extend only out to approximately 12 kpc. Across the distance from the Sun to 12.4 kpc, the maximum extent of the measurements, the carbon abundance drops at most by a factor of two. Given the lack of data points beyond approximately 12 kpc, the true carbon gradient may substantially differ from the direct extrapolation. Studies of abundances of N, O and Fe in H ii regions in other spiral galaxies have shown a ‘flattening’ of the metallicity gradient in the outer regions^42^. If such dampening of the metallicity decline applies to the Milky Way, as studies of open clusters suggest^42^, it would certainly change the extrapolated gradient. Detailed modelling of the gas-phase elemental abundances in the outer disks of galaxies has yet to be developed^42^ and warrants more comprehensive investigation, as underscored by our molecular observations in WB89-621.

## Methods

### Observations

All molecular spectra were obtained using the ARO 12 m telescope or the IRAM 30 m telescope, located on Kitt Peak, Arizona, and Pico Veleta, Spain, respectively. Observations at the ARO 12 m telescope were taken with a dual-polarization receiver with sideband-separating mixers. Image rejection was typically greater than 18 dB. The intensity scale (*T*_A_^*^) for both the ARO 12 m telescope and IRAM 30 m telescope, determined by the chopper wheel method, is related to the main-beam brightness temperature, *T*_R_, by *T*_R_ = *T*_A_^*^/*η*_B_, where *η*_B_ is the main-beam efficiency (0.82 and 0.81, respectively). The ARO 12 m telescope observations were conducted at 2 mm with the ARO Wideband Spectrometer as the backend, configured to a frequency resolution of 156 kHz and 1 GHz bandwidth, per polarization. The IRAM measurements were taken with the Eight Mixer Receiver at 3 mm in dual-polarization mode, with the FTS 200 (fast Fourier transform spectrometer with a resolution of 200 kHz). The line parameters are provided in Table 1. Total integrations times required for the new identifications were 28.6 h at the ARO 12 m telescope and 17.4 h at the IRAM 30 m telescope, respectively.

### Analysis

The column densities were calculated using the non-local thermodynamic equilibrium radiative transfer code RADEX^43^. The program employs the Sobolev approximation to produce line profiles to compare with measured spectra. RADEX varies the molecular column density (*N*_tot_), gas kinetic temperature (*T*_k_) and the H_2_ gas density (*n*(H_2_)). The data files, which consist of the energy levels, transitions and collisional rate information for each molecule, were obtained from the Leiden Atomic and Molecular Database^44^. Because only two rotational transitions of PN were measured, only two parameters could be varied at a time. The gas kinetic temperature was set to 25 K, a value determined by other molecules that had multiple transitions, such as CH_3_OH and CH_3_CN. The H_2_ density was varied between 1 × 10^5^ and 1 × 10^7^ cm^−3^ and the column density was varied from 1 × 10^9^ to 1 × 10^14^ cm^−2^. Based on the two transitions of PN that were measured, a gas density of *n*(H_2_) of approximately 1.5 × 10^5^ cm^−3^ was determined, which was subsequently used to model the single rotational line observed for PO. The ‘best fit’ was determined through a reduced *χ*^2^ analysis.

## Online content

Any methods, additional references, Nature Portfolio reporting summaries, source data, extended data, supplementary information, acknowledgements, peer review information; details of author contributions and competing interests; and statements of data and code availability are available at 10.1038/s41586-023-06616-1.

## Data Availability

All data is available within the paper, and the references therein, which support the findings of this study. Any additional information may be obtained from the corresponding author.
